# Integrative bioinformatics analysis reveals miR-494 and its target genes as predictive biomarkers of trastuzumab-resistant breast cancer

**DOI:** 10.1186/s43046-020-00028-2

**Published:** 2020-04-03

**Authors:** Adam Hermawan, Herwandhani Putri

**Affiliations:** 1 Laboratory of Macromolecular Engineering, Department of Pharmaceutical Chemistry, Faculty of Pharmacy, Universitas Gadjah Mada Sekip Utara II, Yogyakarta, 55281 Indonesia; 2 Cancer Chemoprevention Research Center, Faculty of Pharmacy, Universitas Gadjah Mada Sekip Utara II, Yogyakarta, 55281 Indonesia

**Keywords:** miR-494, Chemoresistance, Trastuzumab, Bioinformatics, Predictive biomarker

## Abstract

**Background:**

The focus of trastuzumab resistance biomarkers in recent decades has been on epigenetic and non-coding RNA-based mechanisms. In this study, the potential of miR-494 and its target genes as predictive biomarkers for breast cancer (BC) resistance to trastuzumab was identified. The microarray data were obtained from the GEO database, including GSE101841, GSE75669, and GSE66305. Data processing was conducted using GEO2R to obtain differentially expressed genes (DEGs).

**Results:**

The data analysis using GEO2R revealed that DEGs from GSE101841 and GSE75669 consisted of 3 and 135 upregulated miRNAs, respectively. On the other hand, the same analysis revealed 8 and 226 downregulated miRNAs for DEGs from GSE101841 and GSE75669, respectively. A Venn diagram showed that one miR was detectable in serum and tissue samples, namely miR-494. The miR-494 target was predicted using the miRecords database and resulted in 69 target genes. A Venn diagram between miR-494 target genes from miRecords and the mRNA array from GSE66305 revealed three potential targets of *CNR1*, *RBM39*, and *ZNF207*. A Kaplan–Meier survival plot showed that BC patients with a high miR-494 level and a low *ZNF207* mRNA level had significantly worse overall survival. Validation of target genes in BC samples and trastuzumab-resistant and -sensitive BC cells with GEPIA and ONCOMINE highlighted the potential of *CNR1*, *RBM39*, and *ZNF207* as predictive biomarkers of trastuzumab resistance in BC cells.

**Conclusion:**

Taken together, these results suggest that miR-494 plays a role in the mechanism of BC resistance to trastuzumab by involving its target genes *CNR1, RBM39*, and *ZNF207*.

## Background

Human epidermal growth factor receptor 2 (HER2)-positive breast cancer occurs in about 20–30% of breast cancer patients [1]. Trastuzumab, a humanized monoclonal antibody, has been widely used in breast cancer treatment of HER2-positive breast cancer subtypes [2]. However, about 70% of breast cancer patients do not respond to trastuzumab due to de novo resistance [2].

The mechanism of trastuzumab resistance involves several biological processes. A review article by Menyhart et al. in 2015 discussed trastuzumab resistance predictive biomarkers in HER2-positive breast cancer, including changes in trastuzumab binding to its receptors and increased ERBB signaling [3]. The same research group stated that increased downstream HER2 signaling (such as PIK3CA, PTEN, SRV, and mTOR), resistance to antibody-dependent cellular cytotoxicity (FcgR), and miRNA are involved in trastuzumab mechanism [3]. In addition, the focus of trastuzumab resistance biomarkers in recent decades has been on epigenetic and non-coding RNA-based mechanisms [4]. Cancer cells that are responsive to trastuzumab will generally develop resistance within 1 year [6]; therefore, the development of biomarkers of trastuzumab resistance is required to achieve better efficacy of trastuzumab treatment in breast cancer.

A review article by Mao in 2016 discussed miRNA candidates as trastuzumab resistance biomarkers from biopsy samples [5]. In addition, miRNA and mRNA biomarkers from serum samples were also developed without obtaining a biopsy of tumor tissue [8]. However, not all serum miRNA is derived from tumor tissue [6]. Collectively, it is important to develop biomarkers from miRNA that is expressed in tumor and serum.

miR-494 plays a pivotal role in cancer progression either as a tumor suppressor or as an oncogene. As a tumor suppressor, miR-494 suppresses breast cancer progression by targeting PAK1 [7]. On the other hand, as an oncogene, miR-494 increases proliferation and migration in MDA-MB 231 breast cancer cells [8]. *CNR1*, *RBM39*, and *ZNF207* are target genes of miR-494. *CNR1* encodes the cannabinoid receptor 1, which mediates the endocannabinoid system and plays a pivotal role in the biological processes of pain, inflammation, cancer, obesity, and diabetes [9]. *RBM39B* encodes RNA binding protein 39, a protein involved in alternative splicing of RNA [10]. *ZNF207* encodes a zinc finger protein 207, a member of the zinc finger protein family that is involved in many biological processes, such as regulation, DNA repair, and cancer progression and metastasis, and can interact with DNA, RNA, and other proteins [11]. The role of those target genes will be explained later in this study.

In this study, using a bioinformatics approach, we retrieved the microarray data of breast tumor tissue samples that originated from Hispanic, Caucasian, and Asian ethnic groups. In addition, we used miRNA microarray data from tissue and serum samples and found one miRNA that was detectable in serum and tumor tissues, namely miR-494. The miR-494 targets were sought and compared with mRNA microarray data from patients who were resistant to trastuzumab. The prognostic values of miR-494 and the target genes as well as target gene expression (*CNR1, RBM39,* and *ZNF207*) in BC samples were evaluated using Kaplan–Meier survival curves and GEPIA. Confirmation of the reliability of the target genes in trastuzumab-sensitive and -resistant BC cells was conducted using ONCOMINE. The genetic alterations in selected genes were analyzed using cBioPortal database. Generated results are highlighting biomarkers from serum and tumor tissues that are statistically reliable for predicting trastuzumab chemoresistance in patients with heterogeneous characteristics.

## Methods

### Data collection and processing

The microarray data were obtained from the GEO database. Data processing was conducted using GEO2R, an online tool for GEO data analysis based on the R programming language (https://www.ncbi.nlm.nih.gov/geo/geo2r/). Differentially expressed genes (DEGs) between trastuzumab-sensitive and -resistant patients were screened. An adjusted *P* value < 0.05 and IlogFCI > 1 were used to select significant DEGs. A Venn diagram was prepared to identify DEGs from GSE101841 and GSE75669 using InteractiVenn (http://www.interactivenn.net/index2.html) [12].

### miRNA target prediction

The miR-494 target was predicted using the miRecords database (http://c1.accurascience.com/miRecords/) [13]. Target genes predicted from at least four datasets were selected. A Venn diagram to identify DEGs from GSE66305 and miR-494 target genes from miRecords was prepared using InteractiVenn (http://www.interactivenn.net/index2.html) [12]. The interaction between miR-494 and its target genes at the target sites was analyzed by TargetScan (http://www.targetscan.org) [14], using the default parameter.

### Kaplan–Meier survival analysis

The prognostic value of miR-494 and the target genes (*CNR1, RBM39,* and *ZNF207*) was evaluated using Kaplan–Meier survival curves (http://kmplot.com) and the log-rank test. *P* < 0.05 was selected as the cutoff value [15]. The prognostic value of miR-494 was generated from Kaplan-Meier plotter breast cancer miRNA using a dataset from METABRIC consist of 93 breast cancer patients with HER2 positive. The Kaplan-Meier plot of CNR1, RBM39, and ZNF207 was generated from the KMPlotter breast cancer database. The number of samples was indicated in each plot.

### Validation of target genes in breast cancer samples and trastuzumab-resistant and -sensitive breast cancer cells

Target gene expression in breast cancer samples was validated using GEPIA (http://gepia.cancer-pku.cn), and *P* < 0.05 was selected as the cutoff value [16]. Confirmation of the reliability of the target genes in trastuzumab-sensitive and-resistant breast cancer cells was conducted using ONCOMINE (https://www.oncomine.org), a cancer microarray database, and web-based data-mining platform [17]. Briefly, the expression levels of *CNR1, RBM39,* and *ZNF207* among trastuzumab-resistant breast cancer samples were retrieved from ONCOMINE. The study by Neve et al. (2006) was chosen for further analysis [18].

### Analysis of genetic alterations among the hub genes

The genetic alterations in selected genes were analyzed using cBioPortal (http://www.cbioportal.org) [19]. In the present study, the genes (*CNR1, RBM39*, and *ZNF20*) were screened for genetic alterations in all breast cancer studies available in the cBioportal database. The breast cancer study with the greatest number of genetic alterations was chosen for connectivity analysis.

## Results

### Data collection and processing

**Fig. 1:**
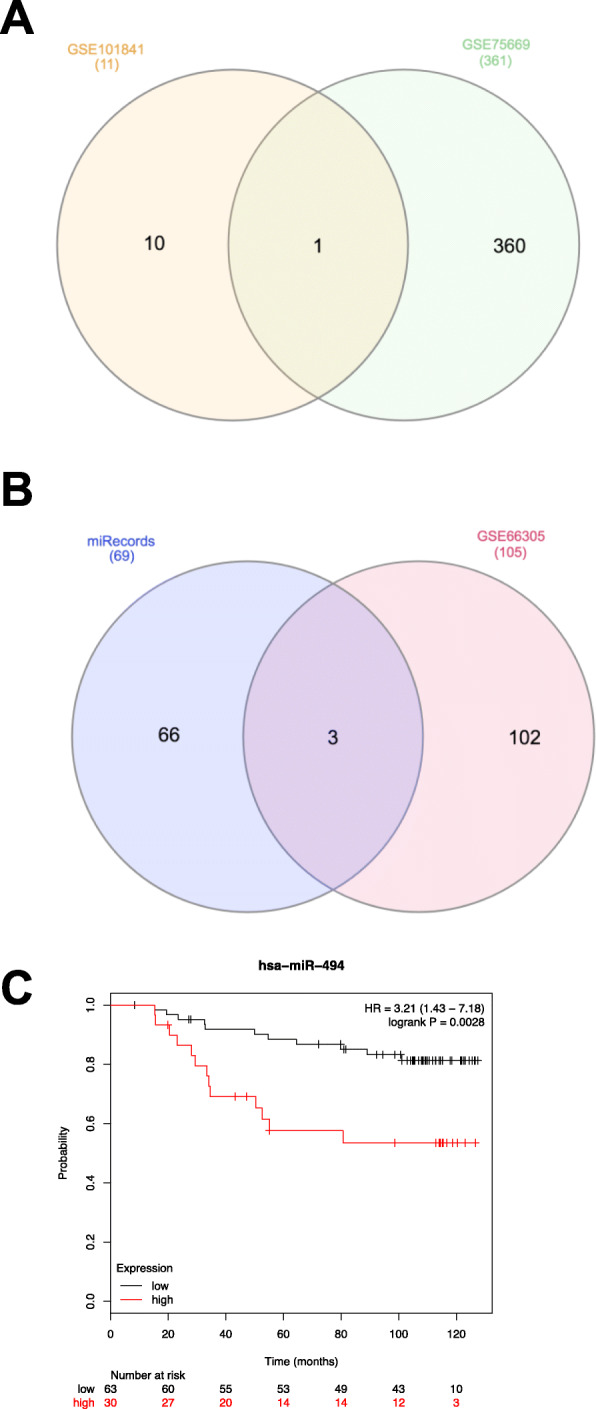
Venn diagram of DEGs from **a** GSE101841 and GSE75669, **b** miRecords and GSE66305, and **c** Kaplan–Meier survival curve related to miR-494 level in the breast cancer samples

We selected three datasets from GEO database (Table 1) based on availability of patient status (complete or partial response), regimen therapy (adjuvant chemotherapy and trastuzumab dose), patient information (race, old, stage of disease), and source of RNA samples (serum or tumor tissue). The data analysis using GEO2R revealed that DEGs from GSE101841 and GSE75669 consisted of 3 and 135 upregulated miRNAs, respectively. On the other hand, the same analysis revealed 8 and 226 downregulated miRNAs for DEGs from GSE101841 and GSE75669, respectively (Supplementary Tables 1 and 2). A Venn diagram showed that one miR was detectable in serum and tissue samples (Fig. 1a). The DEGs from GSE66305 consisted of 40 and 65 upregulated and downregulated mRNAs, respectively (Supplementary Table 3). The miR-494 target prediction using miRecords revealed 69 target genes predicted from at least four databases (Supplementary Table 4). A Venn diagram generated three genes from miRecords and GSE66305, including *CNR1*, *RBM39*, and *ZNF207* (Fig. 1b). The interactions between miR-494 and its target genes at the target sites were analyzed by TargetScan (Supplementary Fig. 1).

**Table 1:** Description of GSE datasets

Accession code	Sample	Number of samples	Type of RNA	References
GSE101841	Serum of tastuzumab-treated patients in China	61 sensitive to trastuzumab, 42 resistance to trastuzumab	miRNA	[20]
GSE75669	Breast tumor from Mexican patients treated with trastuzumab	4 samples from patient with complete response, 4 samples from patients with partial, progressives and poor response to trastuzumab	miRNA	[21]
GSE66305	Breast tumor from Italian patients treated with trastuzumab	5 samples from patients with complete response, and 18 patients with partial response to trastuzumab	mRNA	[22]

### Kaplan–Meier survival analysis

**Fig. 2:**
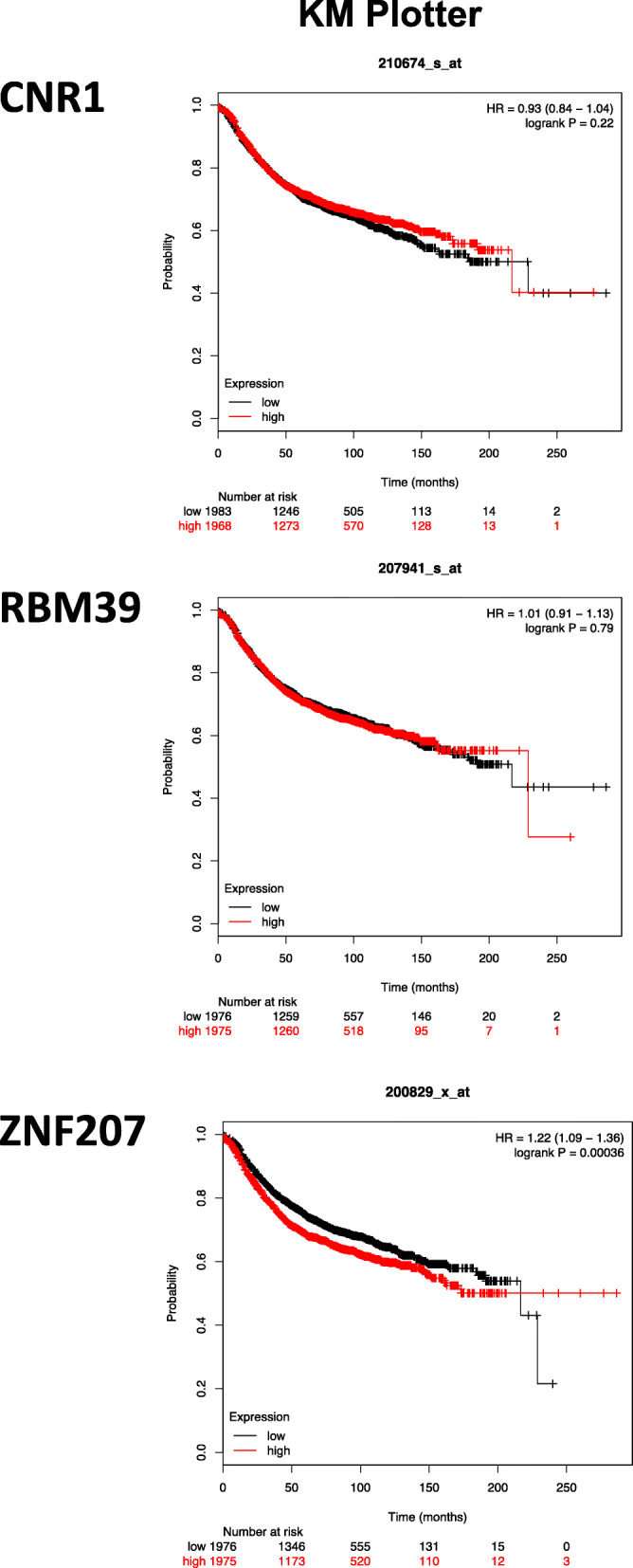
Overall survival curve of breast cancer patients expressing *CNR1, RBM39,* and *ZNF207,* as analyzed by KM Plotter

The Kaplan–Meier plot showed that patients with high miR-494 levels had significantly worse overall survival than those in the low expression level group (*P* = 0.0028, Fig. 1c). Overall survival was also obtained according to the low and high expression levels of each target gene. The results showed that patients with a high *ZNF207* mRNA level (*P* = 0.00036) had better survival than patients with a low mRNA level (Fig. 2). Moreover, patients with high *CNR1* (*P* = 0.22) and *RBM39* (*P* = 0.79) mRNA levels had a worse survival than those with low mRNA levels, although these results are not statistically significant (*P* > 0.05).

### Validation of target genes in breast cancer samples and trastuzumab-resistant and -sensitive breast cancer cells

**Fig. 3:**
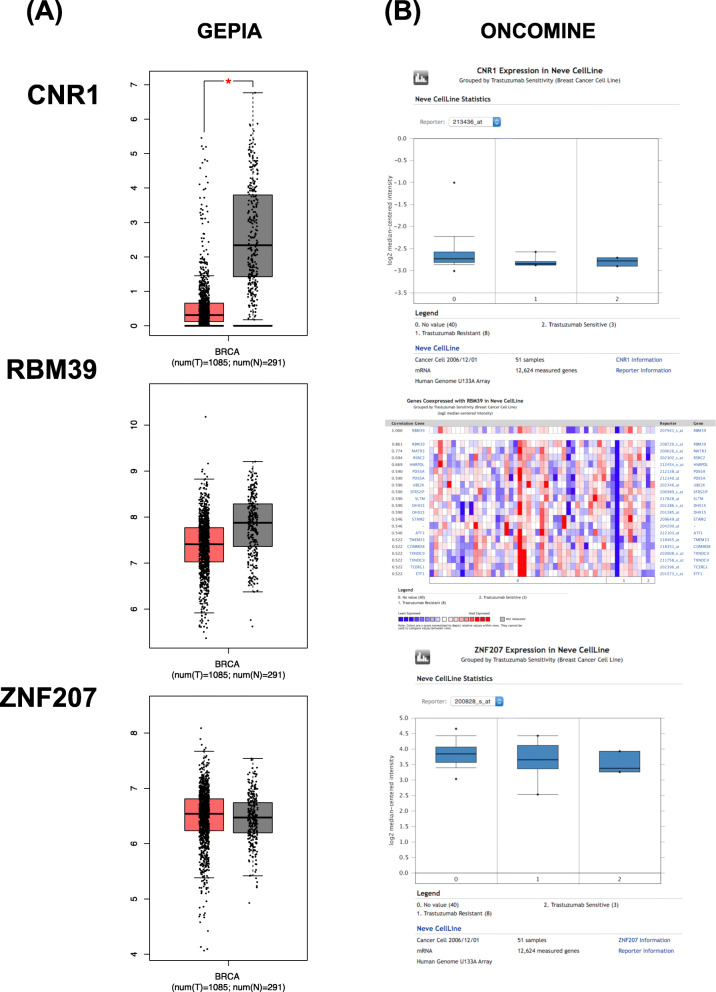
*CNR1, RBM39,* and *ZNF207* mRNA levels in **a**. Breast cancer samples, analyzed by GEPIA and **b** Trastuzumab resistance breast cancer samples, analyzed by ONCOMINE

Gene expression validation using GEPIA showed that the *CNR1* mRNA level was significantly lower in patients with breast cancer than in those without (Fig. 3a). Moreover, no significant differences in *RBM39* or *ZNF207* mRNA levels were observed between the normal and breast cancer samples. Validation of target gene expression using ONCOMINE showed that the *CNR1* mRNA level in trastuzumab-resistant breast cancer cells was lower than that in trastuzumab-sensitive breast cancer cells (Fig. 3b). Moreover, the *RBM39* and *ZNF207* mRNA levels were not different between trastuzumab-resistant and -sensitive breast cancer cells.

### Analysis of genetic alterations in the target genes

**Fig. 4:**
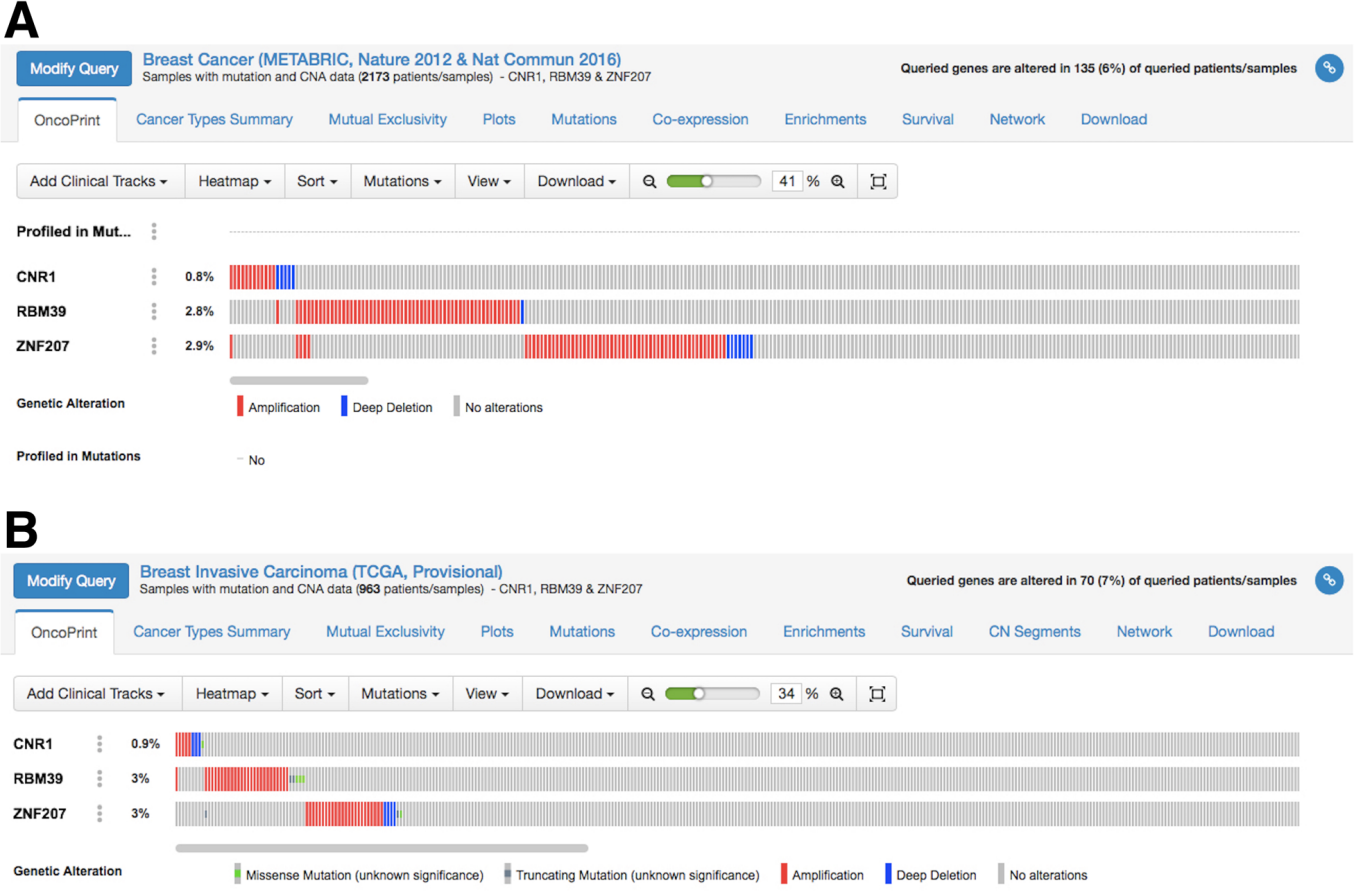
Summary of alterations in *CNR1, RBM39,* and *ZNF207* across breast cancer patients using a study from a METABRIC and **b** TCGA provisional, as analyzed by cBioportal

The genetic alterations in the selected genes were analyzed with cBioPortal using breast samples from METABRIC and the TCGA Provisional study. An oncoprint analysis of the METABRIC samples revealed genetic alterations in *CNR1*, *RBM39*, and *ZNF207* in 0.8%, 2.8%, and 2.9% of breast cancer samples, respectively (Fig. 4a). Moreover, the oncoprint analysis of the TCGA provisional samples showed genetic alterations in *CNR1*, *RBM39*, and *ZNF207* in 0.9%, 3%, and 3% of the breast cancer samples, respectively (Fig. 4b).

**Fig. 5:**
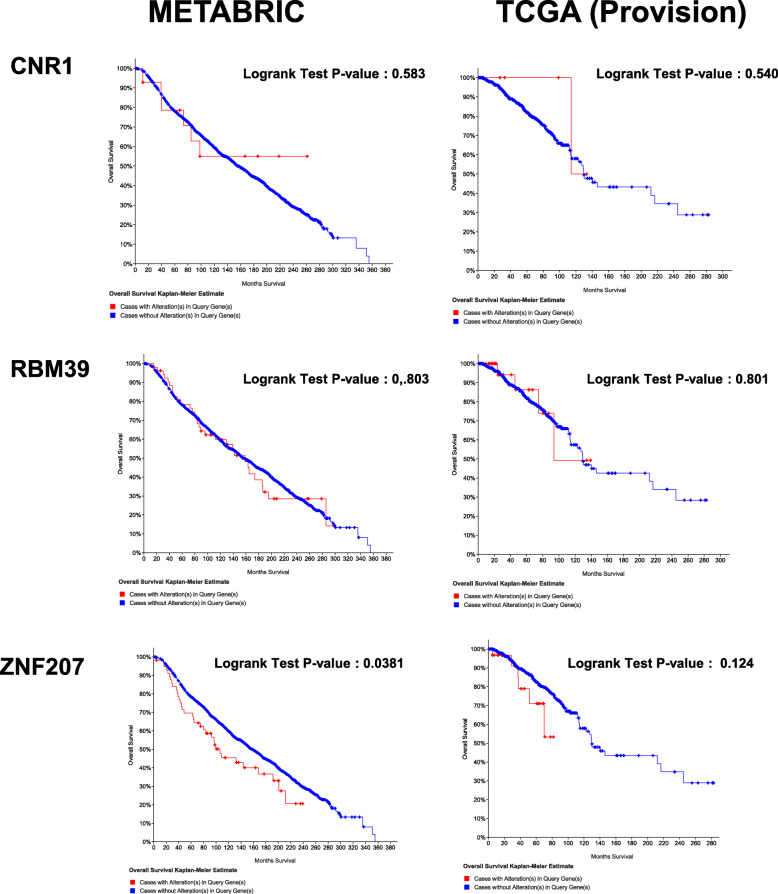
Overall survival related to the genetic alterations in *CNR1, RBM39,* and *ZNF207* across breast cancer patients using a study from METABRIC and TCGA (Provision), analyzed by cBioportal

The Kaplan–Meier survival plots revealed that the METABRIC breast cancer samples with genetic alterations in *ZNF207* had significantly worse overall survival than those in the no change *ZNF207* group (*P* = 0.0381, Fig. 5). No significant difference was observed in the genetic alterations related overall survival plot of *CNR1* and *RBM39* in the METABRIC samples, as well as *CNR1*, *RBM39*, and *ZNF207* in the TCGA provisional samples.

**Fig. 6:**
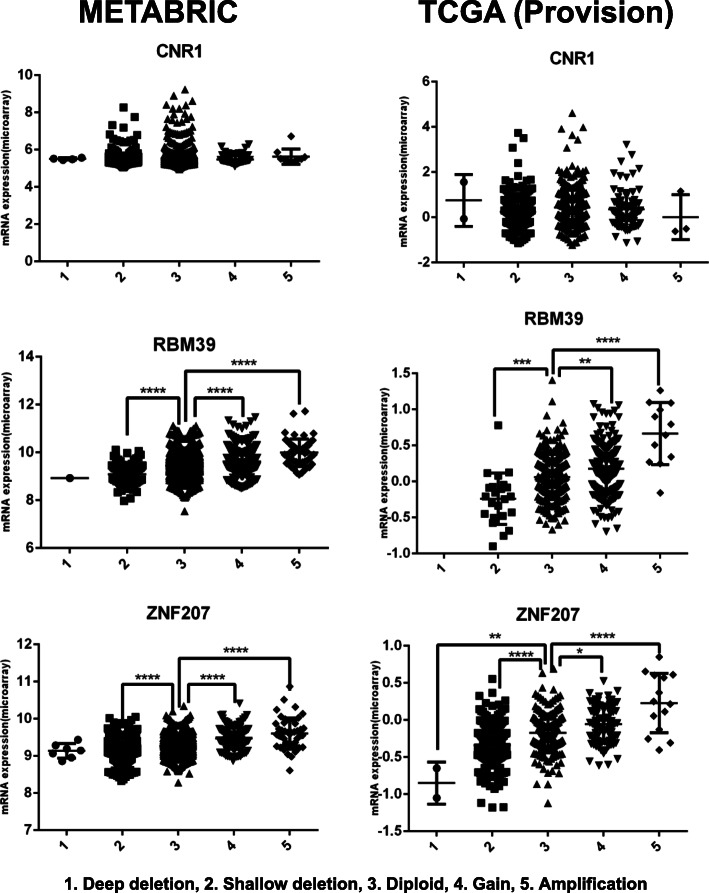
Copy number alterations for *CNR1, RBM39,* and *ZNF207* across breast cancer patients using a study from METABRIC and TCGA (Provision), analyzed by cBioportal. Statistical analysis was conducted using Student’s *t* test. *or **or ***or ****indicates *P* < 0.05 or *P* < 0.01 or *P* < 0.001 or < 0.0001, respectively

The mutation count and *CNR1* mRNA expression was not obvious in the METABRIC and TCGA samples, respectively (Fig. 6). In the METABRIC samples, *RBM39* and *ZNF207* mRNA expression levels were lower in cases with shallow deletion, and higher in cases with gain and amplification than that in the diploid (without change). In the TCGA samples, the *RBM39* mRNA expression was lower in cases with deep and shallow deletion, and higher in cases with gain and amplification than that in the diploid (without change). In the TCGA samples, *ZNF207* mRNA expression was lower in cases with deep and shallow deletion and higher in cases with gain and amplification.

## Discussion

Trastuzumab resistance is a major problem that hinders the effectiveness of trastuzumab in patients with HER2-positive breast cancer. This study aimed to identify predictive biomarkers of trastuzumab resistance in breast cancer patients. Although research on this biomarker has been widely carried out, the clinical trial results have been controversial. Therefore, we need a study that combines several biomarkers as predictors to obtain targeted therapy.

This study was designed to find a predictive biomarker for trastuzumab resistance in serum and tissue samples. miRNA dysregulation is a possible mechanism of trastuzumab resistance; therefore, one of the therapeutic strategies to overcome resistance is manipulating the regulatory network [23]. The results of this study revealed that miR-494 was detectable in serum and breast cancer tissue and is a potential predictive biomarker of trastuzumab resistance in breast cancer (Fig. 1a).

miR-494 can act either as a tumor suppressor gene. miR-494 inhibits breast cancer progression by targeting PAK1 in breast cancer cells [7] and increases proliferation and migration in MDA-MB 231 breast cancer cells [8]. miR-494 also inhibits the cancer stem cell phenotype and reverses lapatinib resistance by downregulating FGFR2 in HER2 gastric cancer patients [24]. Moreover, miR-494 upregulation decreases cell viability, induces apoptosis in MCF-7, and MDA-MB-231 breast cancer cells by targeting nicotinamide phosphoribosyltransferase (NAMPT) [25]. On the other hand, miR-494 also acts as an oncogene. miR-494 increased proliferation and migration in MDA-MB 231 breast cancer cells [8]. Further validation of the miR-494 role in HER2-positive breast cancer cells and the trastuzumab resistance mechanism is warranted.

The results of this present study revealed three miR-494 target genes, namely *CNR1*, *RMB39*, and *ZNF207* (Fig. 1b). CNR1 acts as either a tumor suppressor gene or as an oncogene. CNR1 acts as a tumor suppressor gene in human cervical carcinoma cells [26]. In breast cancer cells, CNR1 tends to play a role as a tumor suppressor gene by inhibition of the cell cycle in human breast cancer cells [27]. However, the role and mechanism of CNR1 in breast cancer resistance to trastuzumab is poorly understood. RBM39 regulates splicing during erythropoiesis [10]. Overexpression of RBM39 is associated with a poor prognosis in patients with myeloma [28]. In addition, the same research group stated that binding of RBM39 impedes the interaction between RBM39 and E3 ubiquitin ligase and subsequently prevents RBM39 degradation [28]. Nevertheless, the role and mechanism of RBM39 in trastuzumab resistance is an interesting topic for further study. *ZNF207* overexpression increases and is a poor prognostic marker in patients with hepatocellular carcinoma [29]. The fusion of HER2 ZNF207 promotes trastuzumab resistance in gastric cancer cells due to the inability to bind to trastuzumab [30]. Further investigation of ZNF207 role in breast cancer resistance to trastuzumab needs to be conducted.

The results of the present study show that a higher level of miR-494 and *ZNF207* is significantly associated with low overall survival (Figs. 1c and 2). Validation of target genes in breast cancer samples and trastuzumab-resistant and -sensitive breast cancer cells with GEPIA and ONCOMINE (Fig. 3) highlighted the potential of CNR1, RBM39, and ZNF207 as predictive biomarkers of trastuzumab resistance in breast cancer cells. Moreover, genetic alterations in ZNF207 were associated with overall survival in the cBioportal (Fig. 5).

One of the advantages of this study is that selected biomarker candidates can be applied to patients with various ethnic characteristics. In this present study, we retrieved microarray data from trastuzumab-resistant breast cancer patients of various ethnicities, namely Asian, Caucasian, and Hispanic. miR-494 can be detected in serum samples and tumor tissues; therefore, it could be easily applied in the clinic because a tissue biopsy is not necessary. Nevertheless, the negative regulation of miR-494 against CNR1, RBM39, and ZNF207 has not been demonstrated; therefore, further validation is required to develop miR-494 and target genes (*CNR1*, *RBM39*, and *ZNF207*) as predictive biomarkers of breast cancer resistance to trastuzumab.

## Conclusion

In conclusion, this present study highlighted miR-494 and its target genes *CNR1, RBM39,* and *ZNF207* as key genes and predictive biomarkers of breast cancer resistance to trastuzumab. Further study to develop miR-494 as a predictive biomarker of breast cancer resistance to trastuzumab is required.

## Supplementary information

**Additional file 1: Figure S1.** miR-494-target gene interactions, analyzed by TargetScan.

**Additional file 2: Table S1.** DEGs from GSE101841.

**Additional file 3: Table S2.** DEGs from GSE75669.

**Additional file 4: Table S3.** DEGs from GSE66305.

**Additional file 5: Table S4.** The miR-494 target prediction, analyzed using miRecords.

## Data Availability

All data generated or analyzed during this study are included in this published article [and its supplementary information files].

## Abbreviations

BC: Breast cancer
CNR1: Cannabinoid receptor 1
DEGs: Differentially expressed genes
FC: Fold change
HER2: Human epidermal growth factor receptor 2
miR: Micro RNA
RBM39B: RNA binding protein 39
ZNF207: Zinc finger protein 207
