# Supplementary figures and images for: Integrative bioinformatics analysis reveals miR-494 and its target genes as predictive biomarkers of trastuzumab-resistant breast cancer
Source: J Egypt Natl Canc Inst. 2020 Apr 3;32:16. doi: 10.1186/s43046-020-00028-2 (PMC13317115; doi:10.1186/s43046-020-00028-2)

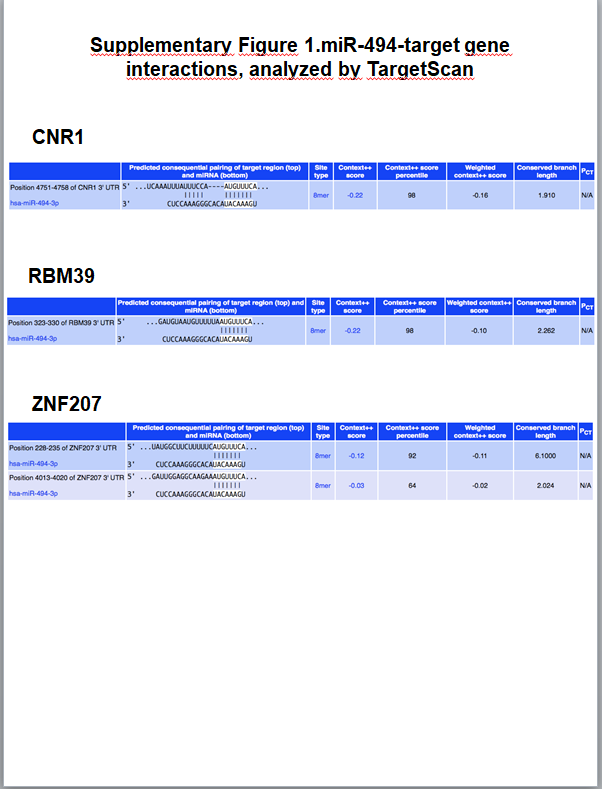

Supplement: Supplementary file 1 — Additional file 1: Figure S1. miR-494-target gene interactions, analyzed by TargetScan. [file 43046_2020_28_MOESM1_ESM.docx]
